# Increased shape and size offerings of femoral components improve fit during total knee arthroplasty

**DOI:** 10.1007/s00167-014-3163-6

**Published:** 2014-07-16

**Authors:** Yifei Dai, Giles R. Scuderi, Charles Penninger, Jeffrey E. Bischoff, Aaron Rosenberg

**Affiliations:** 1 Zimmer, Inc., PO Box 708, Warsaw, IN 46581-0708 USA; 2 Insall Scott Kelly Institute, 210 East 64th Street, New York, NY 10065 USA; 3 Rush University Medical Center, 1725 W. Harrison St., Suite # 1063, Chicago, IL 60612 USA

**Keywords:** TKA, Femoral component, Morphological fit, Overhang

## Abstract

**Purpose:**

Contemporary total knee arthroplasty femoral component designs offer various degrees of fit amongst the global population. The purpose of this study was to assess component fit of contemporary femoral component design families against multiple ethnicities.

**Methods:**

Using a multi-ethnic dataset including Caucasian, Indian, and Korean subjects, this study investigated component fit in six contemporary femoral component design families (A: *Persona*™, B: *NexGen* ^®^, C: *Sigma* ^®^, D: *GENESIS*™ II, E: *Triathlon* ^®^, F: *Vanguard* ^®^). Component overhang/underhang was measured between the resected distal femur and its corresponding component size and compared across design families and ethnicities. The severity of overhang/underhang and propensity of downsizing due to clinically significant overhang were quantified for the overall dataset and each ethnicity.

**Results:**

In all the overhang cases, Designs A and B had significantly lower component overhang than the other designs (*p* < 0.02). In all the underhang cases, Designs C and E had significantly greater underhang than the other designs (*p* < 0.01). Component design influenced the occurrence (% bones) of component downsizing due to clinically significant overhang (>3 mm), with the highest incidence observed in Designs D (20.5 %) and F (17.7 %), and the lowest incidence observed in Designs A (0 %) and B (0.4 %). Variation in component fit was significantly impacted by designs (*p* < 0.01) but not ethnicities (n.s.).

**Conclusions:**

The inclusion of multiple ML/AP shape offerings and the increased number of available sizes in Design A, as compared to other contemporary femoral component design families studied, result in improved femoral component fit across various ethnicities.

## Introduction

Accurate alignment, proper bone cuts, and good soft tissue balancing are all key components that determine the short- and long-term success of total knee arthroplasty (TKA) surgery [27]. However, achieving these goals may require use of a femoral component oversized in the mediolateral (ML) dimension, with associated ML overhang on the femoral bone. Component overhang within unicompartmental knees has been shown to lead to worse patient outcomes at 5 years post-operatively [6]; and it has been suggested that component overhang accounts for 27 % of all incidences of clinically relevant knee pain after TKA [19]. In the review of conditions that may cause painful TKA, Dennis et al. [10] pointed out that intra-articular soft tissue impingement due to TKA component overhang can result in distal femoral osteophytes, extruded bone cement, intra-articular fibrous bands, and painful irritation of the knee tendons and ligaments. In order to avoid soft tissue impingement caused by component overhang, the femoral component may be downsized. However, a downsized femoral component that is too small in the anterior–posterior (AP) dimension can cause laxity in flexion; balancing of the flexion and extension gaps then requires over-resection of the distal femur to elevate the joint line, leading to inferior clinical outcome [1, 12]. As such, reducing the incidence of overhang through the use of femoral implants with anatomically based AP/ML ratio and sizing is important to the clinical performance of TKA.

Numerous morphologic studies have demonstrated high variability in the size and shape of the distal femur [15, 18, 24, 25, 28]. In a 2012 study, Mahfouz et al. showed ethnic differences in the aspect ratio, AP dimension, and patellar groove size of the distal femur. This study, along with several other investigations, reported that Asian population are generally smaller in distal femoral size and have a different aspect ratio compared to the Caucasian population [18, 24, 25, 28]. Furthermore, statically significant differences in distal femoral morphology have also been found within Asian ethnicities [15]. Researchers have suggested that the design of TKA component should consider ethnic differences to better fit the knees [25].

Many contemporary TKA implant systems are intended to be used on the global population. There is thus the need for evaluating the morphological fit of contemporary femoral component designs across the global population, to ensure component shapes and sizes are comprehensive. In a 2003 study, Hitt et al. [14] have shown that the ML sizing of contemporary TKA femoral component designs tends to be too large for smaller knees and that the designs assessed do not account for the aspect ratio changes across bone size. Studies have compared contemporary femoral component design families against Asian anatomy and reported mismatches in both size and aspect ratio [7, 13]. In a study on Chinese knees, Cheng et al. [7] reported that two out of five TKA systems used in China were oversized in the ML dimension and only one component family accounted for the change in aspect ratio across sizes, but the rate of change did not fully reflect that of the Chinese anatomy. Assessment on the fit of contemporary designs in Korean population has suggested that contemporary femoral component designs have a tendency towards under-coverage in small femora and overhang in large femora [13]. Though these studies suggest that contemporary femoral component designs may not fully accommodate morphological variability across global populations, they were not well tuned to surgical parameters specific to each design system. For example, the dimensions of multiple femoral component designs were compared to distal femoral resections not directly related to individual designs [7, 13, 14]; similarly, the impact of surgical techniques on the resection was not considered [13, 14]. Additionally, assessments were often based on manual measurements of the surgical resections, thus introducing user variability [13, 14].

In this present work, we developed a set of improved methodologies for quantifying the morphological fit of contemporary femoral TKA designs to multi-ethnic distal femur anatomy. In particular, the study measured component fit across a multi-ethnic dataset spanning Asian and Caucasian subjects, utilizing a fully automated algorithm to properly size and resect distal femora based on a specific surgical referencing philosophy and design-specific surgical resection parameters without requiring manual user intervention. The aim was to evaluate the ability of contemporary TKA designs to match the anatomy of a diverse multi-ethnic patient population, and the impact of ethnicity and design factors (shape and size) on the fit. It was hypothesized that increased shape (ML/AP ratio) and size offerings in TKA femoral component designs will improve their morphological fit to the resected femur.

## Materials and methods

### Bone data

A total of 277 healthy right femora were selected from a dataset that spans a wide range of sizes and includes both Asian and Caucasian ethnicities. A demographic information summary of the subjects is given in Table 1. Asian subjects were recruited from Indian and Korean clinics following ethical approval and informed consent from each patient. CT scans of the lower extremity were performed using consistent imaging resolution (pixel size 0.75–0.85 mm, slice distance 1 mm). Caucasian data were derived from CT scans of dry bones (pixel size 0.63 mm, slice distance 0.63 mm). All subjects were pre-screened to rule out moderate or severe deformities, osteophytes, and former trauma to the bones. Digital surface models (Unigraphics, Siemens PLM Software, Plano, TX) of the femora were created through segmentation of the CT scans. A set of anatomical landmarks relative to TKA surgery were annotated automatically and approved by experienced users on each femur using the *ZiBRA*™ Anatomical Modeling System, a proprietary software platform with advanced capabilities for digital orthopaedic morphological analysis [3].

**Table 1:** Demographic information for the subjects studied

Subject	Gender	*N*	Age (years, mean ± SD)	Stature (m, mean ± SD)
Indian	Male	36	53.6 ± 7.3	1.68 ± 0.06
Indian	Female	38	54.8 ± 7.0	1.56 ± 0.06
Korean	Male	34	62.3 ± 7.9	1.69 ± 0.05
Korean	Female	34	58.9 ± 7.1	1.56 ± 0.05
Caucasian	Male	63	50.8 ± 10.7	1.77 ± 0.07
Caucasian	Female	72	65.3 ± 13.1	1.61 ± 0.08

### Femoral component designs

Digital three-dimensional models of six contemporary TKA femoral component design families from various manufacturers were assessed in this study: (1) Design A: *Persona*™ The Personalized Knee System (Zimmer, Warsaw, IN); (2) Design B: *NexGen* ^®^ Complete Knee Solution (Zimmer, Warsaw, IN); (3) Design C: *Sigma* ^®^ Knee Solutions (DePuy Synthes, Warsaw, IN); (4) Design D: *GENESIS*™ II Total Knee System (Smith and Nephew, Memphis, TN); (5) Design E: *Triathlon* ^®^ Knee System (Stryker, Kalamazoo, MI); and (6) Design F: *Vanguard* ^®^ Complete Knee System (Biomet, Warsaw, IN) (Table 2). Designs A and B have multiple ML size offerings for a specific component AP size. Design A has both standard and narrow ML offerings per AP size, with the finest increment (2 mm) selection in AP sizes amongst all the design families. Design B offers standard and gender sizes in ML widths, each comes with standard and minus sizes in AP dimension. Designs C–F have single ML offerings across component AP size. All available sizes and ML offerings in the design families were included in this study.

**Table 2:** Femoral component design families used in this study

Design	*A*	*B*	*C*	*D*	*E*	*F*
# AP sizes	12	11*	7	8	8	9
AP increments (mm)	2**	2	3–5	3–4	3–4	2–3
# ML size offerings per AP	1–2	1–2	1	1	1	1
Aspect ratio (ML/AP)	1.0–1.3	1.0–1.2	1.1–1.2	1.2–1.3	1.1–1.2	1.1–1.2
Frontal view						
Sagittal view						

* Standard sizes and minus sizes

** For size 1–11

### Component conformity to resected femur

**Fig. 1:**
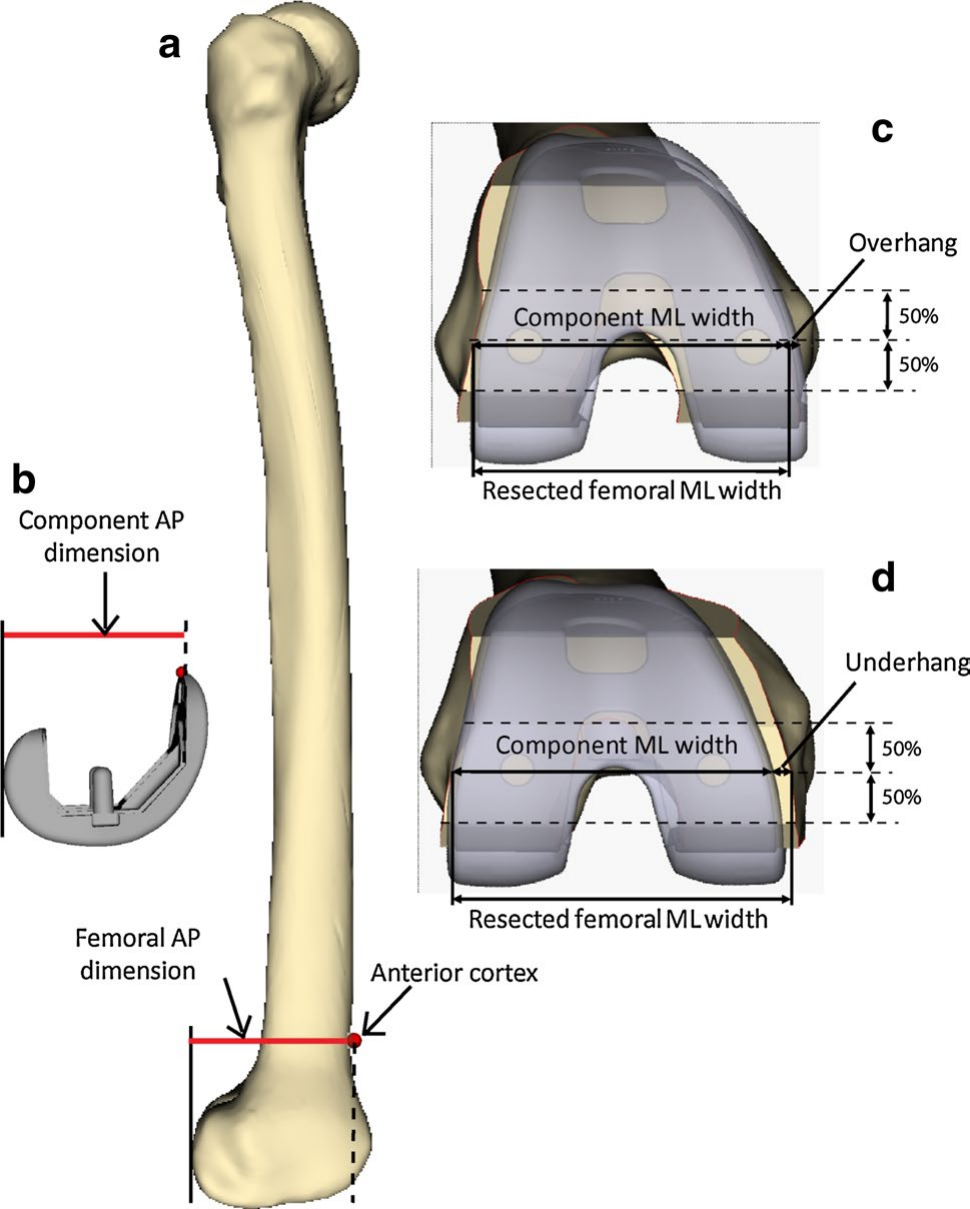
Measurements of AP dimension on the **a** distal femur and **b** femoral component. A representative femur with virtual TKA resection and femoral component placement is also shown demonstrating measurements of **c** overhang, **d** underhang, and **c**, **d** component and resected femoral ML widths

In *ZiBRA*™ System, the AP dimension was digitally and automatically measured on the femora as the projected length from the anterior cortex of the distal femur to the plane tangent to the posterior condyles (Fig. 1a). A corresponding measurement was also performed on the femoral components (Fig. 1b). For a given design family, each femur was sized by a computational algorithm, which selected the component size that most closely matched but did not exceed the femoral AP dimension. Virtual TKA resection was then performed on the distal femur based on the specific design and size selected, in accordance with an anterior referencing technique confirmed by two board-certified orthopaedic surgeons (GRS and AR). The resections restored the original joint line and ensured accurate rotational alignment of the anterior and posterior cuts relative to the transepicondylar axis [16, 21]. Varus/valgus alignment was set to be perpendicular to the femoral mechanical axis, with flexion/extension alignment perpendicular to the anatomical axis of the distal femur. A cartilage thickness of 2 mm was assumed to account for distal and posterior condyle cartilage [8, 19]. All resections were visually approved by experienced users. Each individual femoral contour following resection was exported for further analysis (MATLAB, Mathworks, Natick, MA).

In addition to AP dimension, the ML width of the resected femur was measured midway between the anterior and posterior borders of the distal cut plane (Fig. 1c, d). To describe the shape of the distal femur, the aspect ratio was calculated as the ML/AP ratio for each bone. The metrics were compared to the dimensions and aspect ratio on the femoral components, measured similarly as resected femur (Fig. 1c, d). The femoral ML width was correlated with component size (component AP dimension) and evaluated across design families. For each specific design, an overhang incidence bound was defined as the minimum ML width of the resected femora that fit the component size plus 3 mm, based on a previous study that reported overhang of more than 3 mm approximately doubles the incidence of clinically important knee pain 2 years after TKA surgery [19]. Any design that has no ML size offering below the overhang incidence bound indicates incidence of more than 3 mm overhang is unavoidable for the dataset unless AP conformity is compromised. The aspect ratio of the femora was regressed against the aspect ratio of their properly sized components per design, with a regression slope of 1 indicating a perfect aspect ratio match between the design and the resected femora. The closeness of the data to an ideal fit for each design was calculated as the root mean squared deviation (RMSD) of the deviations between the femora and components. Higher RMSD reflects poor fit of the design to the dataset due to mismatch in the aspect ratio, leading to surgical compromise.

### Incidence and severity of component overhang

For each design, the differences in ML width between the resected femora and their associated component sizes were calculated. Overhang or underhang was identified if the femoral ML width was smaller or larger than the ML width offering of the matching femoral component (Fig. 1c, d), with >3 mm overhang defined as clinically significant, indicating a requirement of downsizing. The amount and incidence of overhang in general, as well as incidence of downsizing, were analysed across all designs and compared between ethnicities.

### Institutional review board approval

The Asian CT scans in this study were collected from live patients. Each CT data collection has been approved by the institution to which the study principle investigators were primarily affiliated. The following listed the names of the institutions that granted the approval:

1. *Korean CT data:* Department of Radiology, Asan Medical Center, Seoul, South Korea.
2. *Indian CT data:* Sant Parmanand Hospital, New Delhi, India.

### Statistical analysis

The arithmetic mean, standard deviation, and distribution of the measurements were determined. One-way analysis of variance (ANOVA) tests were performed to compare measurements across ethnicities and designs (Minitab, Minitab Inc., State College, PA). The null hypothesis was that all the ethnic or design group means are equal; the level of significance was defined at *p* = 0.05.

## Results

### Component conformity to resected femur

**Fig. 2:**
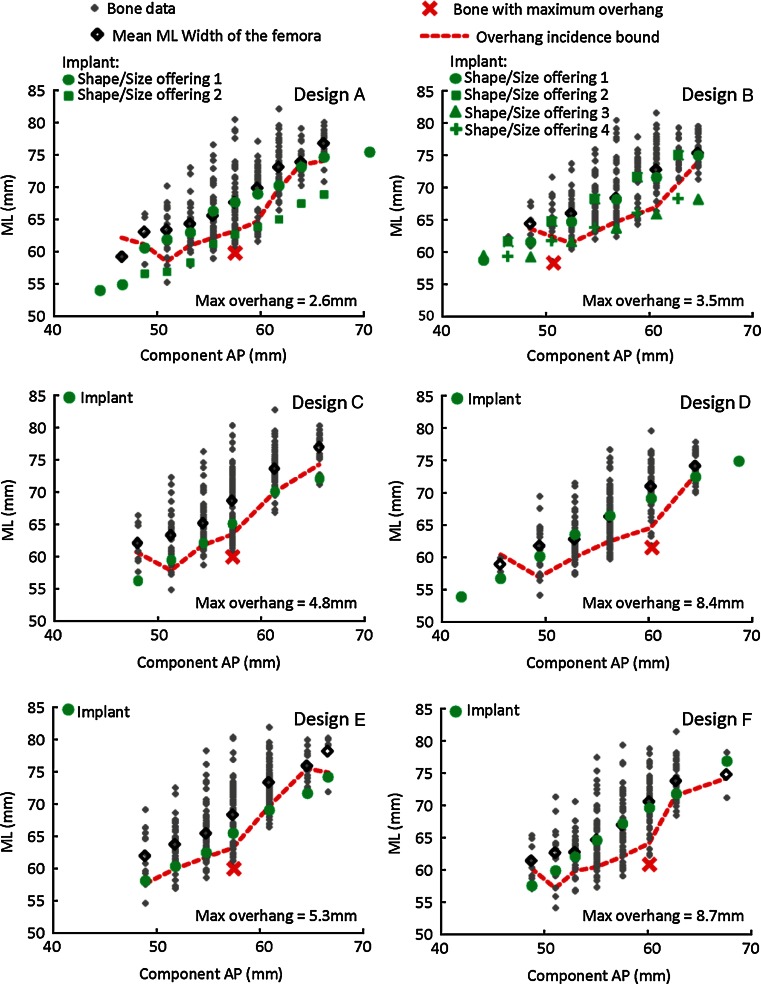
Correlation between resected femoral ML widths and the size of the component (component AP dimension) for each design family, overlaid with associated component dimensions. The overhang incidence bound denotes the threshold for the smallest component ML required to fit all the bones in the dataset without a single case of clinically significant overhang and represents an offset of 3 mm from the bone with smallest ML dimension for a given AP. For a given AP size, if a component is not available with an ML width below the overhang incidence bound, then excessive overhang will be realized. The bone with the highest component overhang was identified for each design, with maximum overhang amount indicated

The multiple ML offerings in Design A enabled proper fit to all the bones in the dataset across component AP sizes without a single case of clinically significant overhang (constant availability of component ML size below the overhang incidence bound for the entire dataset) (Fig. 2). Similarly, for Design B, the multiple ML size offerings provided adequate ML size choice for the majority of the bones, expect for a slightly increased incidence of clinically significant overhang in one component AP size (57 mm). Designs C–F had oversized ML width in most of their component AP sizes, indicating unavoidable clinically significant overhang exist in the dataset. Across ethnicities, for the femora with component overhang, Designs A and B had significantly lower overhang amount than Designs C–F (Table 3; *p* < 0.02). For the femora with component underhang, Designs C and E had significantly greater underhang amount than the other designs (*p* < 0.01).

**Table 3:** Percentages of the femora that have either over- or underhang, with the over- and underhang amounts, respectively

	Design family					
	*A*	*B*	*C*	*D*	*E*	*F*
% Bones with component overhang	7.5	6.5	16.2	44.8	20.9	40.1
Overhang amount (mm) mean ± SD	1.2 + 0.7*	1.0 + 0.8^a^	1.9 + 1.3^b^	3.0 + 2.1^c^	1.9 + 1.4^b^	3.1 + 2.2^c^
% Bones with component underhang	92.5	93.5	83.8	45.2	79.1	59.9
Underhang amount (mm) mean ± SD	−3.2 ± 2.4^a^	−3.4 ± 2.5^a^	−4.7 ± 3.1^b^	−3.5 ± 2.7^a^	−4.8 ± 3.1^b^	−3.4 ± 2.8^a^
Pooled ML difference (mm) mean ± SD	−2.9 ± 2.5^a^	−3.0 ± 3.3^a^	−3.7 ± 3.8^b^	−0.6 ± 4.0^C^	−3.2 ± 4.5^ab^	−0.6 ± 4.6^C^

Pooled differences between component and bone ML dimensions are also indicated

Positive values indicate overhang; negative values indicate underhang

^abc^Indicate statistical differences

**Fig. 3:**
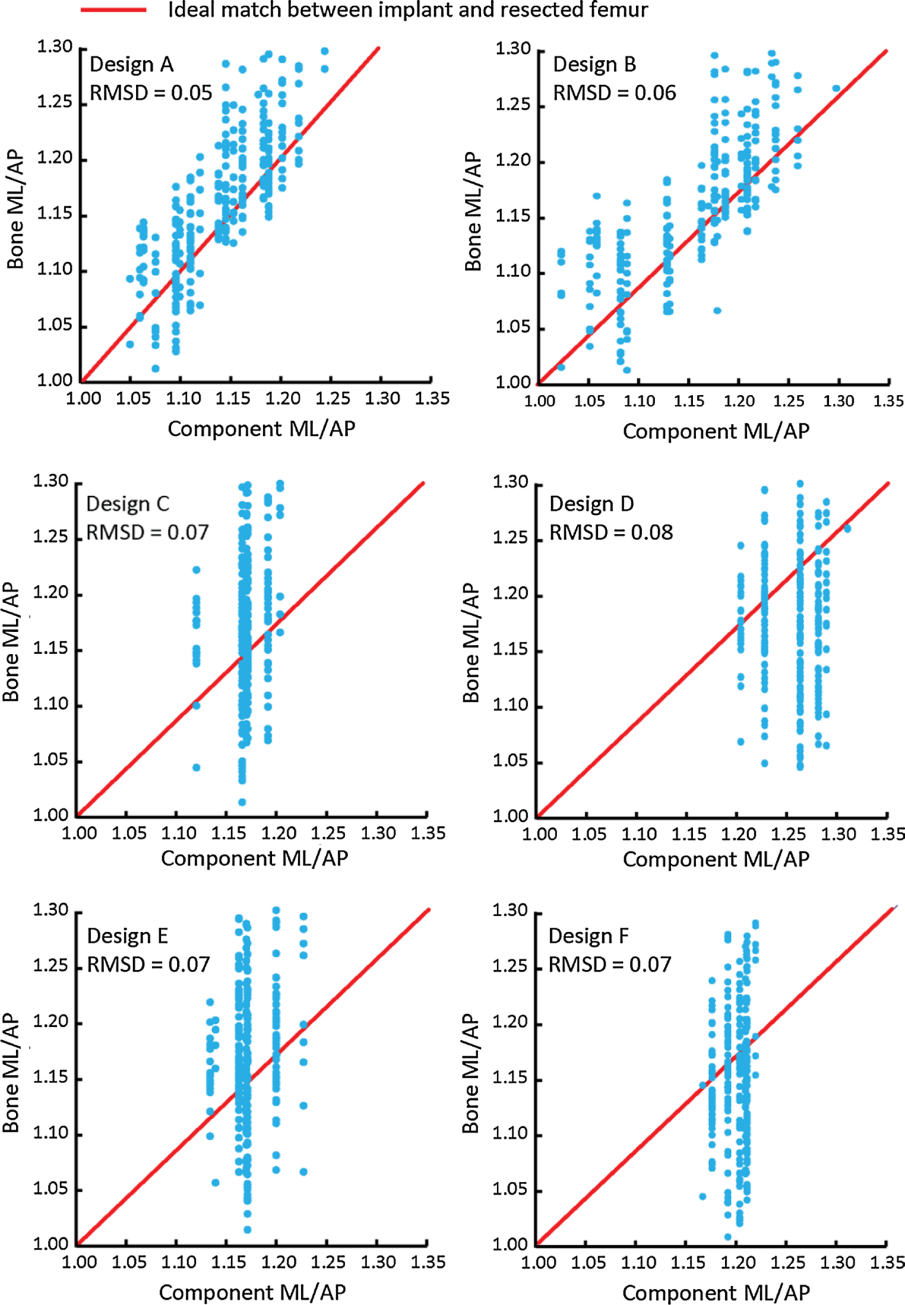
Correlation between femoral and component aspect ratios for each design family. Closer proximity to the ideal match line (correlation slope = 1, indicating perfect match between component and bone ML) indicates better fit. Design A was found to have the closest match with the ideal component shape amongst all six designs evaluated compared to other designs

Designs A and B both captured the shape variability in the resected femur (Fig. 3). In contrast, Designs C–F had greater deviation from the aspect ratio of the resected femur, indicating higher incidence of large overhang and/or underhang. The lowest RMSD in aspect ratio was found in Design A (0.05), followed by Design B (0.06), Designs C, E, F (0.07), and Design D (0.08).

### Incidence and severity of component overhang

**Fig. 4:**
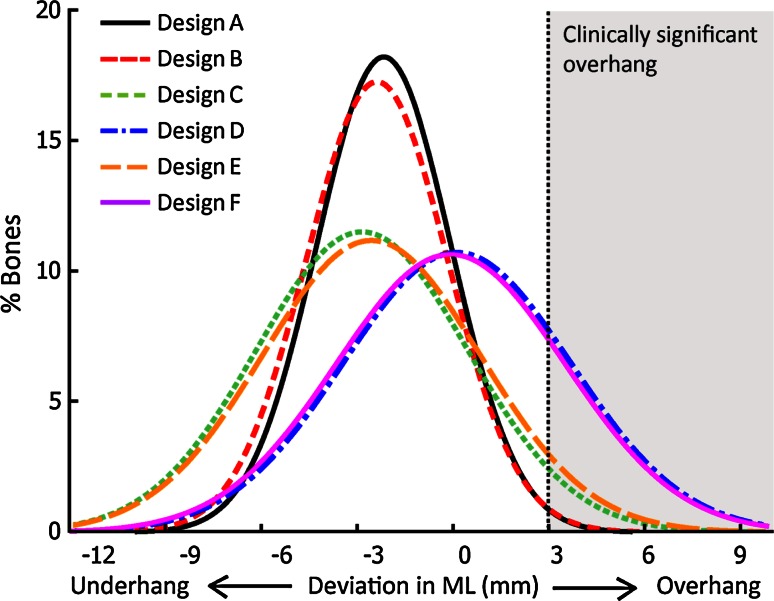
Distribution of overhang/underhang for each component family

Three distinct levels of component fit were observed amongst the designs (Fig. 4). Designs D and F had the highest incidence and severity for clinically significant overhang, followed by Designs C and E (which also had the largest bias towards underhang). Designs A and B exhibited the lowest incidence and severity of clinically significant overhang and had at least 35 % less variability in component-to-bone ML mismatch (SD: 2.2–2.3 mm) than the other design families (Designs C–F, SD: 3.5–3.7 mm).

 A summary of the incidence of downsizing for each ethnicity and design is given in Table 4. The percentage of femora that required downsizing was the highest in Designs D (20.5 %) and F (17.7 %), followed by Designs C (3.2 %) and E (5.4 %). In contrast, none or minimal downsizing was required in Designs A (0 %) and B (0.4 %). Component design significantly impacts the variation in component fit (*p* < 0.01), with significant differences in the incidence of downsizing between designs (*p* < 0.02, Table 4). In contrast, ethnicity did not significantly impact the variation (n.s.). Specifically, Designs D and F consistently exhibited the highest incidence of downsizing across all three ethnicities (12.2–26.5 % of the femora). Compared to the significant differences found between designs, no significant difference was found in downsizing with respect to ethnicity, with average incidences (across designs, per ethnicity) of 8.6, 5.7, and 8.8 % of the femora of Caucasian, Indian, and Korean subjects (n.s.), respectively.

**Table 4:** Percentages of the femora that need to be downsized due to clinically significant overhang (>3 mm) for each ethnicity and design family

% Femora needing downline	Design family						Mean ± SD
	*A*	*B*	*C*	*D*	*E*	*F*	
Caucasian	0.0	0.0	3.7	20.7	7.4	20.0	8.6 ± 9.5
Indian	0.0	1.4	2.7	14.9	2.7	12.2	5.7 ± 6.3
Korean	0.0	0.0	2.9	26.5	4.4	19.1	8.8 ± 11.2
Mean ± SD	0.0 ± 0.0^a^	0.5 ± 0.8^a^	3.1 ± 0.5^b^	20.7 ± 5.8^c^	4.8 ± 2.4^b^	17.1 ± 4.3^c^	
Pooled	0.0	0.4	3.2	20.5	5.4	17.7	

^abc^Indicate statistical differences

**Fig. 5:**
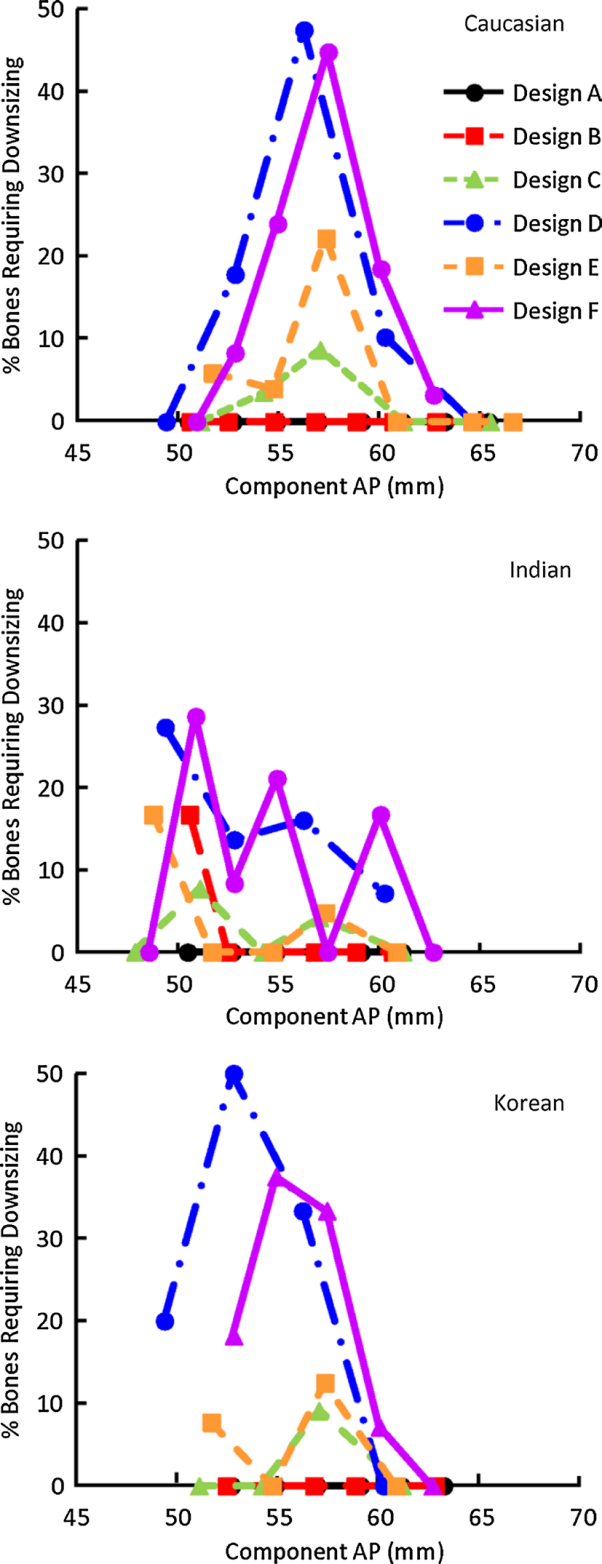
Percentage of femora that require downsizing per design for Caucasian, Indian, and Korean subjects, plotted across component AP size

The highest incidences of downsizing were in mid-sized Caucasian femora and small to mid-size Korean femora in Designs D and F (Fig. 5). Smaller but potentially clinically impactful incidences of downsizing (2.7–7.4 % of the femora) were found in Designs C and E, mostly in small to mid-sized Caucasian and Korean femora, and small Indian femora. Only 1.4 % of the femora required downsizing for Design B, all found in small-sized Indian bones. No downsizing was required in Design A across the ethnicities.

## Discussion

The most important finding of the present study was that contemporary femoral components are generally biased towards component underhang and exhibit wide variations in morphological fit to the distal femur. It also suggests that compared to ethnicity, component design has a greater impact on the variability of femoral component fit and the incidence of component downsizing, with similar performance across the ethnicities investigated for a specific design family. The most noticeable improvement in fit was found in design families with multiple ML offerings per AP size (Designs A and B), as they provide more component selections to match the variability in the distal femur aspect ratio than designs with single ML offerings (Designs C–F). This finding agrees with a recent study, which concluded that designs with multiple ML offerings for a given component AP size may improve component-to-bone fit and reduce the propensity of greater than 2-mm component overhang/underhang in Chinese patients [29]. Amongst all six design families investigated, Design A exhibited no incidence of downsizing and had the smallest deviation in aspect ratio compared to the resected femur. Another contributing factor to the improved fit in Designs A and B may be that they provide more AP size offerings (11–12 AP sizes) compared to Designs C–F (7–9 AP sizes). The better component fit observed in Design A over Design B may be due to finer increments in AP sizing (2 mm). Amongst the four designs with a single ML size offering (Designs C–F), although Design F provides the highest number of size offerings, it does not offer improved component fit compared to the designs with fewer size offerings (Designs C and E). The relatively lower incidence of downsizing and less severity of overhang found in Designs C and E may due to their generally lower component aspect ratios compared to Designs D and F.

Good morphological fit between TKA components and the resected knee anatomy is an important factor for success in TKA. Specifically with regard to the femur, AP and ML mismatches are often encountered during the surgical implantation of the femoral component. Several clinical studies have documented component overhang with resulting negative surgical outcomes due to irritation of the soft tissue or overstuffing of the joint space and associated compromise of range of motion [5, 19]. The clinical prevalence of component overhang has been found to be more than 50 % [5], and more than 40 % of the TKA component implantations were reported to have ≥ 3-mm overhang [19]. Mahoney et al. [19] have reported that the presence of femoral component overhang of 3 mm or greater was associated with a 90 % increase in the risk of having clinically important pain following TKA comparing to knees with less than 3-mm overhang. In another study, it was found that overhang of the femoral component can be directly associated with post-operative pain and reduced overall function and flexion angle [5]. Downsizing of the femoral component is often performed as the compromise in order to avoid overhang. However, reducing the size of the component can result in either anterior femoral notching or more commonly reduction of the posterior condylar offset with possible flexion instability [2] regardless of anterior- or posterior-referencing surgical techniques. In addition, Hitt et al. [14] pointed out that undersizing of the femoral component could leave cancellous bone exposed, which may be a source for post-operative bleeding or may be an instigating site for osteolysis when wear debris is present.

Numerous morphologic studies have shown that the dimensions of the distal femur are highly variable and suggested that contemporary femoral component designs may not accommodate morphological differences across ethnicities [7, 13, 14, 18, 25]. These investigations focused on analysis of ethnic or gender variability in femoral anthropometrics only, or comparison of either intact or resected distal femur with component dimensions without consideration of surgical technique and component sizing philosophy. Furthermore, previous assessment on contemporary femoral component designs only focused on either a single ethnicity [7, 13, 29], or did not differentiate ethnicities in the dataset [14]. To our knowledge, this is the first study that automatically evaluated component fit on the resected distal femur across multiple ethnicities spanning Asian and Caucasian subjects, in which sizing and placement following design-specific algorithms. The current analysis provides further insight into the contemporary component fit in global populations.

There are several limitations to this study. First, though resections were specific to each implant system, ideal distal femoral resections were employed for each design; however, clinical variability in resection parameters has been reported in previous studies [20, 22]. Second, each resection utilized an assumed uniform 2-mm cartilage thickness; however, inter-subject, anatomical site-dependent variations in distal femoral cartilage thickness have been documented [4, 11, 23] and shown to be correlated with multiple factors such as age, BMI, loading in the knee, and state of osteoarthritis [4, 11, 17]. Third, all the results here are based on healthy subjects, not TKA candidates. The impact of these limitations on the results may require further investigation.

Although all the measurements performed in this study were based on fully automated computer simulation, the expected resolution of the results is impacted by several aspects of the data pre-processing: (1) accuracy of the automatic annotations of the landmarks depends on the resolution of the CT data, which had sub-millimetre accuracy (up to 2 decimal places); (2) approval of automatically defined landmarks by experienced users introduces some level of inter- and intra-user variability, though this has been shown to be at sub-millimetre level (errors reported in 1 decimal place) [26]; and (3) the surgical reference axes for the distal femoral resection were constructed based on anatomical landmarks and naturally inherited the errors in landmark identification. Based on these considerations, results here were reported at a comparable level of resolution (1 decimal place). Additionally, the accumulated impact of variability from CT data on morphometric analysis of TKA resections has been shown to be within typical clinical bounds of TKA for the workflow utilized here [9], which supports the clinical relevancy of the virtual distal femoral resection in this study.

The clinical implications of the observations in the present study suggest that some contemporary femoral component designs may not accommodate morphological differences across patient populations. This in turn may lead to surgical compromise of femoral bone preparation or clinical complications due to soft tissue impingement, improper balancing of the flexion and extension gaps, and pain. The findings emphasize the importance of properly designing the shape and size of the femoral components to meet the morphological variability of the distal femoral across the global population. Additionally, the results suggest that multiple size and shape offerings can offer improvement of the morphological fit of the femoral components without compromise of soft tissue, joint space balancing, and joint line.

## Conclusion

Varying degrees of morphological conformity across six contemporary TKA femoral designs were found in this study. The data suggest femoral component designs that provide multiple ML/AP shape offerings, and increased number of available sizes can provide necessary component selection to fit the resected femur adequately in the ethnicities investigated without clinically significant overhang.
